# Abdominothoracic Postural Tone Influences the Sensations Induced by Meal Ingestion

**DOI:** 10.3390/nu13020658

**Published:** 2021-02-18

**Authors:** Dan M. Livovsky, Claudia Barber, Elizabeth Barba, Anna Accarino, Fernando Azpiroz

**Affiliations:** 1 Digestive System Research Unit, University Hospital Vall d’Hebron, Centro de Investigación Biomédica en Red de Enfermedades Hepáticas y Digestivas (Ciberehd), Departament de Medicina, Universitat Autònoma de Barcelona, 08193 Bellaterra (Cerdanyola del Vallès), Spain; danlivo@yahoo.com (D.M.L.); claudiabarbercaselles@gmail.com (C.B.); aaccarino@telefonica.net (A.A.); 2 Faculty of Medicine, Hebrew University of Jerusalem, Digestive Diseases Institute, Shaare Zedek Medical Center, Jerusalem 9103401, Israel; 3 Neurogastroenterology Motility Unit, Hospital Clínic, University of Barcelona, 08007 Barcelona, Spain; ebarbaorozco@gmail.com

**Keywords:** meal ingestion, postprandial responses, hedonic sensations, homeostatic sensations, abdominal wall activity, abdominal distension

## Abstract

Postprandial objective abdominal distention is frequently associated with a subjective sensation of abdominal bloating, but the relation between both complaints is unknown. While the bloating sensation has a visceral origin, abdominal distention is a behavioral somatic response, involving contraction and descent of the diaphragm with protrusion of the anterior abdominal wall. Our aim was to determine whether abdominal distention influences digestive sensations. In 16 healthy women we investigated the effect of intentional abdominal distention on experimentally induced bloating sensation (by a meal overload). Participants were first taught to produce diaphragmatic contraction and visible abdominal distention. After a meal overload, sensations of bloating (0 to 10) and digestive well-being (−5 to + 5) were measured during 30-s. maneuvers alternating diaphragmatic contraction and diaphragmatic relaxation. Compared to diaphragmatic relaxation, diaphragmatic contraction was associated with diaphragmatic descent (by 21 + 3 mm; *p* < 0.001), objective abdominal distension (32 + 5 mm girth increase; *p* = 0.001), more intense sensation of bloating (7.3 + 0.4 vs. 8.0 + 0.4 score; *p* = 0.010) and lower digestive well-being (−0.9 + 0.5 vs. −1.9 + 0.5 score; *p* = 0.028). These results indicate that somatic postural tone underlying abdominal distention worsens the perception of visceral sensations (ClinicalTrials.gov ID: NCT04691882).

## 1. Introduction

Abdominal bloating and distention are the major and most bothersome complaints in patients with functional gut disorders, such as functional dyspepsia and irritable bowel syndrome [1]. In the past 20 years several studies have investigated the relation between these tandem complaints [2]

Abdominal bloating, i.e., the sensation of abdominal pressure/fullness, is a subjective sensation of visceral origin. Abdominal bloating in healthy subjects can be induced by experimental increments of gut contents, for instance by inflation of a gastric balloon [3], intestinal gas infusion [4] or a meal overload [5]. Patients with functional gut disorders complaining of bloating have increased visceral sensitivity, so that physiological volumes, well tolerated by healthy subjects, reproduce their customary symptoms [6,7]. Hence, bloating sensation may be induced by large intraluminal loads and/or increased sensitivity of the gut.

It has been consistently shown that patients complaining of abdominal distension exhibit an objective increase in girth [2]. Abdominal distention is a behavioural somatic response featuring diaphragmatic contraction and descent coupled with relaxation and protrusion of the anterior abdominal wall [8,9,10].

The relation between bloating and distention is not clear. In the past both terms were used indistinctively, and this unprecise terminology contributed to the confusion [1]. However, patients clearly distinguish between the subjective sensation of increased abdominal pressure/fullness (i.e., bloating) and visible abdominal distension [11,12,13]. Since patients frequently relate distension to bloating sensation, it could be speculated that the somatic response is a conditioned protective attempt to mitigate the visceral sensation, somewhat analogous to the abdominal contraction covering peritoneal irritation, but the attempt turns out ineffective and the distension becomes then a major complaint. The potential interactions between abdominal bloating and distention are highly relevant for the management of patients in clinical practice, and our specific aim was to determine the effect of abdominal distention on visceral sensation. To this aim, we measured the effect of intentional abdominal distension on experimentally induced bloating sensation in healthy subjects. 

## 2. Material and Methods

### 2.1. Participants

Sixteen healthy, non-obese, non-dieting and weight-stable women without history of gastrointestinal symptoms were recruited by public advertising to participate in the study. Exclusion criteria were chronic health conditions, prior obesity, previous abdominal surgery, use of medications (except occasional use of NSAIDs and antihistamines), history of anosmia and ageusia, current dieting or any pattern of selective eating such as vegetarianism, alcohol abuse and use of recreational drugs. Absence of current digestive symptoms was verified using a standard abdominal symptom questionnaire (no symptom > 2 on a 0–10 scale). Psychological and eating disorders were excluded using the following tests: Hospital Anxiety and Depression scale (HAD), Dutch Eating Behavior Questionnaire (DEBQ—Emotional eating, External eating, Restrained eating), and Physical Anhedonia Scale (PAS). Candidates were asked whether they liked the test meal to be tested (see below) and those who did not were not included.

The research was conducted according to the Declaration of Helsinki. The protocol for the study had been previously approved by the Institutional Review Board of the University Hospital Vall d’Hebron, (Comitè d’Ètica d’Investigació Clinica, Vall d’Hebron Insititut de Recerca; protocol number PR(AG)338/2016 approved 28 October 2016, revised 11 December 2020) and all participants provided written informed consent.

### 2.2. Experimental Design

Single-center study performed at the Vall d’Hebron University Hospital, comparing postprandial digestive sensations (abdominal bloating and digestive well-being) during consecutive maneuvers of diaphragmatic contraction (i.e., descent) versus diaphragmatic relaxation (i.e., ascent) in a cross-over randomized design. Outcomes were the effect of somatic maneuvers on abdominal bloating sensation (primary outcome) and on digestive well-being (secondary outcome). The study protocol was registered at ClinicalTrials.gov (ID: NCT04691882). All co-authors had access to the study data and reviewed and approved the final manuscript.

### 2.3. General Procedure

Participants were instructed to refrain from strenuous physical activity the day prior to the study, to consume only a light breakfast at home after an overnight fast and to report to the laboratory, where the probe meal was administered 4 h after breakfast (only water was allowed until 2 h before the study). Studies were conducted in a quiet, isolated room. First, participants were taught to control the postural tone of the abdominal wall and maintain two different positions for 30-s episodes: (a) diaphragmatic contraction and anterior abdominal wall relaxation (visible abdominal protrusion) and (b) diaphragmatic relaxation and anterior wall contraction (flat abdomen); after a 30-min training period all participants effectively learned the abdominothoracic maneuvers. Second, a probe meal was administered up to maximal satiation in order to induce abdominal bloating sensation. Immediately after ingestion participants were asked to stand up and lean on a high bench in a comfortable position and were instructed to sequentially perform 30-s maneuvers alternating diaphragmatic contraction and diaphragmatic relaxation. Two sequences of 4 alternating maneuvers starting with a different order were performed separated by a 1-min rest interval; in random allocation; half of the participants started with one maneuver and the other half in with the other. Digestive sensations were scored immediately before meal ingestion, every 4 min during meal ingestion, and at the end of each abdominal maneuver in the postprandial period. 

### 2.4. Probe Meal

The probe meal consisted of a mixture of nutrients (Fresubin® Protein Energy—Fresenius Kabi, Germamy; 1.5 kcal/mL) and non-absorbable polyethylenglycol 4000 (27 g/L) to prevent water absorption. The nutrient drink is presented in 3 flavors (chocolate, vanilla and cappuccino) and participants were asked to select their choice. Participants were then instructed to drink the probe meal at a standard rate (75 mL/min) until maximal satiety.

### 2.5. Perception Measurements

Two 10 cm scales graded from −5 to + 5 were used to measure hunger/satiety (extremely hungry/completely satiated) and digestive well-being (extremely unpleasant sensation/extremely pleasant sensation); abdominal bloating-fullness sensation was measured using a 10 cm scale graded from 0 (not at all) to 10 (very much). Subjects received standard instructions on how to fill-out the scales [14].

### 2.6. Ancillary Validation Study

A subset of the participants (n = 6) underwent a second study on a separate day measuring the physiological effects of the somatic maneuvers. Following the same experimental procedure, the following outcomes were measured.

#### 2.6.1. Abdominal Girth

The method has been previously described in detail [15]. Briefly, a non-stretch belt (48 mm wide) was placed over the umbilicus and fixed to the skin on the back to prevent slipping. The overlapping ends of the belt were adjusted carefully by two elastic bands to maintain the belt constantly adapted to the abdominal wall. Girth measurements during the study were taken directly with a metric tape measure fixed to the belt. Measurements were obtained before meal ingestion, immediately after ingestion, and during the somatic maneuvers without manipulation of the belt-tape assembly. Previous studies validated the reproducibility of the measurements and sensitivity of this method to consistently detect the small variations in girth induced by various experimental conditions [15,16,17,18,19,20,21,22,23].

#### 2.6.2. Position of the Diaphragm

In previous studies, we showed that displacement of the diaphragm can be equally evaluated by monitoring the position of either the right liver dome by CT scan or the right lower margin imaged by ultrasonography [4]. As previously described, the position of the lower margin of the right liver lobe at the right anterior axillary line was identified by ultrasonography (Eco 1, Chison Medical Technologies, Jiangsu, China) using a 3.5 MHz curved array transducer held over the edge of the costal wall in a coronal plane with the shaft held in a horizontal position and the head in an axial direction. At each determination (before ingestion, after ingestion and during somatic maneuvers) participants were instructed to breathe normally and the mid-point between the end-inspiratory and end-expiratory position of the liver margin, assessed over a period of six respiratory cycles, was marked on the overlying skin, then the differences between determinations were measured.

#### 2.6.3. Intragastric Pressure and Respiratory Rate

A manometric catheter (Latitude® Esophageal Motility Catheter, Unisensor AG, Attikon, Switzerland, model GIM600E) (2.7 mm outside diameter) with 4 micro-balloons at 5 cm intervals was introduced through the nose and placed with 2 recording sites above and 2 below the gastroesophageal junction at the beginning of the experiments; the balloons were filled with air and intraluminal pressures were continuously recorded. Intrathoracic (esophageal) and intraabdominal (intragastric) pressures and respiratory rate were measured before and after meal ingestion and during each somatic maneuver. 

#### 2.6.4. Heart Rate and Heart Rate Variability (Vagal Tone)

Continuous heart rate monitoring with high quality inter-beat data was recorded during the entire experiment using a Bluetooth heart rate strap (H10, Polar Electro, Kempele, Finland). R-R intervals and cardiac interbit intervals were obtained. Heart rate variability (HRV) was assessed at the beginning of the experiments before and during intubation, before and after meal ingestion, and during each somatic maneuver. Measurements before intubation and before meal ingestion were performed over a period of 5 min; measurements after meal ingestion and during the somatic maneuvers were performed over 30-s periods; the validity of this ultrashort period of HRV analysis has been shown in several setting [24,25,26,27,28,29]. HRV analysis of the exported data was performed on a computer using a dedicated HRV software (Kubios Premium ver. 3.4.2). Prior to HRV computation all IBI data were visually inspected for correctness and then underwent automatic artifact correction to calculate mean values of root mean square of the successive differences between normal heartbeats (RMSSD) as a marker of vagal tone (higher RMSSD reflects higher vagal tone). The RMSSD is obtained by calculating each successive time difference between heartbeats in ms. Each of the values is squared and the result is averaged, then the square root of the total is obtained [30,31,32].

#### 2.6.5. Galvanic Skin Responses (Sympathetic Activity)

Two 11 mm Ag/AgCl dry electrodes were secured on the index and middle fingers of the right hand, with Velcro straps and electrodermal activity (EDA) was continuously recorded (MySignals, Libelium Comunicaciones Distribuidas, Zaragoza, Spain). Galvanic skin responses (GSR) were measured as in EDA phasic changes within 1–5 s following different conditions: intubation, meal ingestion and somatic maneuvers [30]. EDA depends on the activity of sweat glands, and hence, the conductance of electrical signals through the skin; GSR reflect sympathetic activity. 

### 2.7. Statistical Analysis

Statistical analysis was performed using IBM statistics SPSS v25; a significance level of 5% (two tails) was used in all analyses. Descriptive statistics were used to define baseline demographic characteristics of participants. Data are presented as mean values ± standard error. The Shapiro-Wilk Test was used to determine normality of data distribution. Parametric normally distributed data were compared by paired or unpaired Student’s t-test, as corresponded; otherwise, the Wilcoxon signed rank test was used for paired data, and the Mann-Whitney U test was used for unpaired data. The association of parameters was analyzed using Pearson’s R test.

## 3. Results

### 3.1. Demographics and Study Conduction

Participants had a mean age of 30 ± 2 years, 62 ± 3 Kg body weight, 164 ± 2 cm height and 22.5 ± 0.7 Kg/m^2^ body mass index. All participants had a normal bowel habit and scored HAD, PAS, and DEBQ within the normal range. Each study day, participants confirmed compliance with the dietary instructions. All participants completed the studies and were included for analysis.

### 3.2. Responses to Meal Ingestion

**Figure 1:**
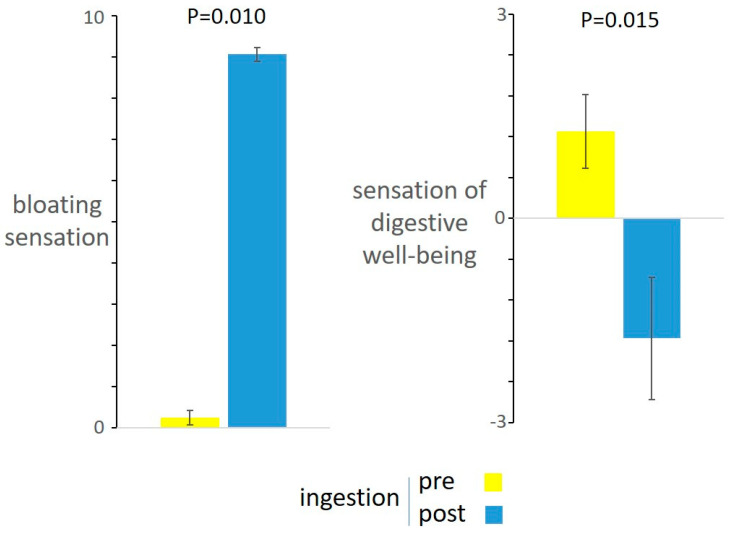
Responses to meal ingestion. The challenge meal induced bloating sensation and impaired sensation of digestive well-being.

Before ingestion, participants were hungry with sensation of digestive well-being. During the ingestion period, baseline hunger declined and the hunger/satiation axis progressively shifted towards maximal satiation by the end of the meal. The amount of food tolerated was 839 ± 29 mL. No significant correlations were found between food tolerance and body mass index (BMI) (*r* = −0.228; *p* = 0.396) or weight (Pearson *r* = −0.212; *p* = 0.430). By the end of the meal, participants experienced abdominal bloating and negative sensation of digestive well-being. (Figure 1).

During the ingestion period in the validation studies, intragastric pressure initially decreased, and then gradually increased, but the changes were not statistically significant. Meal ingestion was associated with a significant increase in girth (by 13 + 3 mm; *p* = 0.009), accent of the diaphragm (by 9 + 4 mm; *p* = 0.057), decrease in vagal tone (by 7.4 ± 1.2 ms RMSSD; *p* = 0.013), increase in heart rate (by 9.6 ± 2.6 beats per min; *p* = 0.014) and respiratory rate (by 3.5 ± 1.3 breaths per min; *p* = 0.047), without significant changes in intrathoracic pressure (0.3 + 0.7 mmHg difference; *p* = 0.767) and sympathetic activity (0.1 + 0.4 µS difference; *p* = 0.817).

### 3.3. Effect of Thoracoabdominal Postural Tone on Postprandial Sensations

**Figure 2:**
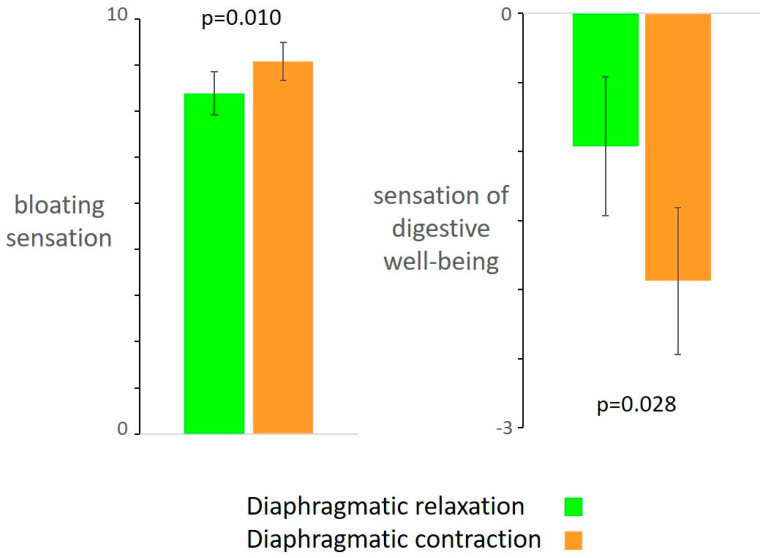
Effect of somatic maneuvers on digestive sensations. Diaphragmatic contraction (i.e., intentional abdominal distension) was associated with increased postprandial bloating and impaired digestive well-being.

Compared to diaphragmatic relaxation, diaphragmatic contraction was associated with more intense sensation of bloating (by 0.7 ± 0.2 score; *p* = 0.010) and lower digestive well-being (by 1 ± 0.4 score; *p* = 0.028). (Figure 2). The order of the maneuvers did not influence the results (no differences were detected between the subjects allocated to either sequence, i.e., initiating with diaphragmatic contraction or relaxation).

### 3.4. Effect of Thoracoabdominal Postural Tone on Physiological Parameters (Validation Study)

As compared to diaphragmatic relaxation, maneuvers of diaphragmatic contraction produced anterior protrusion of the abdominal wall with significant girth increment (by 32 ± 5 mm; *p* = 0.001), diaphragmatic descent (by 21 + 3 mm; *p* <0.001), increase in intragastric pressure (by 8 ± 2 mmHg; *p* = 0.016) and a mild decrease in heart rate (by 3 ± 1 beats per minute; *p* = 0.033) without significant changes in intrathoracic pressures 0.4 ± 0.8 mmHg; *p* = 0.628), respiratory frequency (−0.6 ± 0.4; breaths per minute; *p* = 0.422) and vagal tone (2.5 ± 1.6 ms RMSSD; *p* = 0.195). In contrast to the significant sympathetic arousal produced by nasopharyngeal stimulation during intubation with the manometry catheter (3.6 ± 0.8 µS increment; *p* = 0.004), the somatic maneuvers did not elicit a galvanic response (0.04 ± 0.08 µS difference; *p* = 0.207)

## 4. Discussion

Our data demonstrate that the activity of the abdominal wall influences perception of visceral stimuli, specifically, intentional abdominal distention heightens bloating sensation induced by a meal overload. 

In normal conditions meal ingestion induces physiological responses that accomplish the digestion, as well as homeostatic (satiety, fullness) and hedonic sensations (digestive well-being and improved mood) [33]. Postprandial sensations depend on the characteristics of the meal and the appropriate digestive response, as well as on constitutive and inducible factors of the host, the latter influenced by a variety of conditioning mechanisms [34].

The meal load is determinant in the sensations induced by ingestion; the meal load bears a direct relation with homeostatic sensations and a bimodal relation with hedonic sensations: small comfort meals up to a certain level induce satisfactory homeostatic sensations (satiation, mild fullness, digestive well-being and consummatory reward), whereas larger meals induce aversive fullness sensation [34,35]. In this study we used a challenge meal to induce bloating sensation and dissatisfaction, mimicking postprandial symptoms in patients with functional gut disorders. This experimental model has been previously validated in our laboratory [6], and it has been shown that the homogeneous liquid meal induces stronger homeostatic sensations and less satisfaction than normal mixed meals with similar caloric and volume loads [36].

The intercostal muscles, diaphragm and the anterior abdominal muscles exert phasic rhythmic activity related to respiration (lung ventilation). These phasic contractions are superimposed on a tonic, i.e., sustained, muscular activity (postural tone). Muscular tone in this chain of muscles (intercostals, diaphragm and anterior abdominal wall) is finely coordinated, allowing a physiological redistribution of thoracoabdominal contents [4,37] without affecting respiratory function (tidal volume and the physiological dead space of the lungs): an upwards shift of contents is orchestrated by elevation of the costal wall (increase in intercostal tone), diaphragmatic ascent (decrease in tone) and flat abdomen (tight anterior abdominal wall), so that the abdominal cavity is elongated in cranial direction and the lungs accommodate into the upper chest. Conversely, a caudal shift involves descent of the costal wall (intercostal relaxation), diaphragmatic descent (increase in tone) and anterior wall protrusion (reduced tone). 

As described before (see Introduction), abdominal distention with objective anterior wall protrusion in patients is due to an uncontrolled increase in diaphragmatic tone and blockade of the diaphragm in caudad position with downwards displacement of abdominal contents (abdominophrenic dyssynergia) [6,8,38,39]. Patients can be trained to control abdomino-phrenic postural tone, release the diaphragmatic blockade and correct abdominal distention, but the learning process, based on visual biofeedback signalling of abdominal thoracic muscular activity, is rather complex [8,38]. In contrast to patients, healthy subjects readily learned to master abdomino-phrenic tone and shift the balance back and forth from diaphragmatic relaxation/flat abdomen to diaphragmatic contraction/abdominal distention. In this experimental model we showed that during diaphragmatic contraction perception of gut stimuli is higher than during diaphragmatic relaxation, and intragastric content produced more severe postprandial bloating sensation with impaired digestive well-being.

Several mechanisms may be involved in this effect. We previously showed that sympathetic arousal released by lower body negative pressure (blood sequestration in the lower extremities) increases intestinal sensitivity and heightens perception of intestinal distention [40]. The lack of galvanic response to the somatic manoeuvres in the present study makes this mechanism unlikely; furthermore, no changes in vagal tone and minor changes in cardiorespiratory function were detected. Diaphragmatic contraction produced a relatively small but consistent increase in intragastric pressure. Conceivably, the external compression of the diaphragm over the full stomach heightened perception of intragastric content, in a similar way as abdominal palpation might do. Hence, the exploratory mechanistic outcomes of our study suggest that the somato-visceral interaction is mediated by mechanical factors. 

We wish to acknowledge some limitations of our study. First, we cannot ascertain whether our results are extensible to men, because this proof-of-concept study was performed in women. Women were selected because previous studies showed that they are more susceptible to conditioning of postprandial sensations and probably this may explain the female preponderance of functional digestive disorders [35,41,42]. Second, this study was performed in healthy subjects mimicking the situation in patients with meal-related symptoms and abdominal distention, yet we cannot ascertain to what extent the experimental procedures in healthy subjects reproduced real-life clinical conditions. Third, abdominal symptoms were induced by a meal overload, and it is not clear whether somatic activity may also influence symptoms of a different origin, e.g., colonic distension.

The clinical relevance and importance of the somato-visceral interaction evidenced by our study helps to explain the relation between abdominal bloating and distention in patients with functional gut disorders. It could be speculated that bloating sensation elicits the somatic behavioural response leading to abdominal distention, as a protective mechanism attempting to reduce the visceral sensation; however, our data show that abdominal distention (increased diaphragmatic tone) worsens bloating sensation. Conversely, we have previously shown that correction of abdomino-phrenic dyssynergia by biofeedback effectively prevented abdominal distention [8,38]. That study showed that correction of distension was associated with improvement of digestive symptoms, and our current data explain this unexpected effect.

## 5. Conclusions

Our proof-of-concept study indicates that the somatic postural tone underlying abdominal distention worsens the perception of visceral sensations; these data in healthy subjects help to understand the somato-visceral interactions and the pathophysiological relation of abdominal bloating to distension, two major complaints in patients with functional gut disorders.

## Data Availability

Not applicable.
